# Biological Activities of Deer Antler-Derived Peptides on Human Chondrocyte and Bone Metabolism

**DOI:** 10.3390/ph17040434

**Published:** 2024-03-28

**Authors:** Tsung-Jung Ho, Wan-Ting Tsai, Jia-Ru Wu, Hao-Ping Chen

**Affiliations:** 1 Integration Center of Traditional Chinese and Modern Medicine, Hualien Tzu Chi Hospital, Hualien 970473, Taiwan; tjho@tzuchi.com.tw (T.-J.H.); wanting22@tzuchi.com.tw (W.-T.T.); 2 Department of Chinese Medicine, Hualien Tzu Chi Hospital, Hualien 970473, Taiwan; 3 School of Post-Baccalaureate Chinese Medicine, Tzu Chi University, Hualien 970473, Taiwan; 4 Department of Biochemistry, School of Medicine, Tzu Chi University, Hualien 970374, Taiwan

**Keywords:** chondrocyte, deer antler, Guilu erxian jiao, kyotorphin, osteoblast, osteoclast differentiation

## Abstract

Orally administered “tortoiseshell and deer antler gelatin” is a common traditional medicine for patients with osteoporosis or osteoarthritis. From the pepsin-digested gelatin, we previously isolated and identified the osteoblast-stimulating pentapeptide, TSKYR. Its trypsin digestion products include the dipeptide YR, enhancing calcium ion uptake, and tripeptide TSK, resulting in remarkable 30- and 50-fold increases in mineralized nodule area and density in human osteoblast cells. These peptides were chemically synthesized in this study. The composition of deer antler preparations comprises not only proteins and peptides but also a significant quantity of metal ion salts. By analyzing osteoblast growth in the presence of peptide YR and various metal ions, we observed a synergistic effect of calcium and strontium on the effects of YR. Those peptides could also stimulate the growth of C2C12 skeletal muscle cells and human chondrocytes, increasing collagen and glycosaminoglycan content in a three-dimensional environment. The maintenance of bone homeostasis relies on a balance between osteoclasts and osteoblasts. Deer antler peptides were observed to inhibit osteoclast differentiation, as evidenced by ROS generation, tartrate-resistant acid phosphatase (TRACP) activity assays, and gene expression in RAW264.7 cells. In summary, our findings provide a deep understanding of the efficacy of this folk medicine.

## 1. Introduction

Osteoporosis and osteoarthritis pose significant challenges for the elderly population, with glucosamine supplements commonly used to alleviate their symptoms despite ongoing debates regarding their effectiveness, as reflected in the global market surpassing $1.8 billion US dollars in 2022 [1]. By contrast, traditional Chinese medicine (TCM) usually derives from a variety of natural products [2,3,4], and introduces an intriguing perspective, particularly in the form of “Guilu Erxian Jiao”, also known as “tortoiseshell and deer antlers gelatin (TDAG)”, a prescription frequently administered for joint pain, osteoporosis, and osteoarthritis [5,6,7]. Our recent investigation unveiled a notable penta-peptide, TSKYR, derived from deer antlers within this TCM remedy, showcasing its potential impact on bone health. As this gelatin is administered orally, it can be further broken down into tetra-peptide (P4, SKYR), tri-peptide (P3, TSK), and di-peptide (P2, YR) in the intestine. These peptides have demonstrated a marked ability to stimulate the proliferation of both human osteoblasts and chondrocytes [8].

Notably, both di-peptide YR, known as kyotorphin, and pentapeptide TSKYR, known as neo-kyotorphin, are analgesic peptides discovered in bovine brains [9,10]. More importantly, kyotorphin can trigger calcium ion influx through a G protein-coupled receptor [11,12]. This prompts a fundamental question: Why does TDAG effectively treat osteoporosis? The answer might lie in the enhanced bio-absorption of calcium ions facilitated by di-peptide YR. Furthermore, the tri-peptide TSK plays a crucial role, resulting in a remarkable 30-fold and 50-fold increase in the area and density of mineralized nodule in human osteoblasts [8]. These findings offer solid in vitro evidence supporting the efficacy of using TDAG to treat osteoporosis. The historical utilization of tortoiseshell as a rich, affordable, and viable source of calcium ions becomes apparent, while concurrently, peptides derived from deer antlers enhance the bioavailability of calcium within the human bone system. 

Examining these materials in a comparative context, both tortoiseshell and deer antlers represent rigid animal tissues composed of organic and inorganic components [13]. In Table 1, the mineral composition analysis of human bone, tortoiseshell, and deer antler underscores a notable difference in calcium and strontium content. While human bone showcases higher levels, our exploration into the periodic table reveals both calcium and strontium as alkaline earth metals [14,15,16,17]. The significance of calcium in maintaining human bone health has been extensively documented [18], with strontium also playing a pivotal role in supporting the vertebrate skeletal system [19]. Based on our previous findings demonstrating the activities of the tripeptide TSK in osteoblasts, we proceeded to investigate how deer antler peptides influence mineralized nodule formation in the presence of calcium and strontium ions. Because the muscle and skeletal systems are collectively referred to as the musculoskeletal system in physiology, we also investigated the biological activities of these deer antler peptides in promoting skeletal muscle growth here.

Osteoarthritis, characterized by the progressive degradation of articular cartilage, directs our inquiry into the intricate role played by deer antler peptides in cartilage formation. In tandem with examining their influence on chondrocyte growth, we meticulously assessed their influence on collagen and glycosaminoglycan content, as well as gene expression of chondrocytes, all within three-dimensional environments. Finally, we also investigated the effects of these peptides on the balance between osteoclasts and osteoblasts, fundamental to bone homeostasis.

In this comprehensive exploration, we reveal the multifaceted biological activities of deer antler-derived peptides. From elucidating their effects on bone metabolism to probing their potential role in addressing conditions such as osteoarthritis and osteoporosis, our findings pave the way for novel therapeutic implications. As we traverse historical remedies to contemporary scientific inquiry, the intricate interplay between these peptides and diverse facets of musculoskeletal health becomes apparent.

## 2. Results and Discussion

### 2.1. Effect of Metal Ions on Osteoblast Cell Proliferation

**Figure 1:**
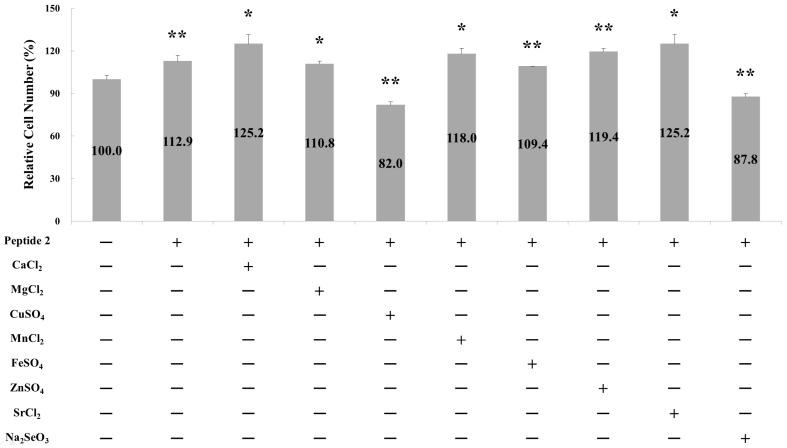
Effects of different metal ions and dipeptide YR on osteoblast growth. Note: Osteoblast proliferation activity is represented as the cell number. Data are presented as means ± SD from three independent experiments. Significance was determined through a one-tailed test analysis, with * *p* < 0.05 and ** *p* < 0.005 indicating a significant difference between peptide 2 combined with different metal ions and the no-treatment condition.

Our previous results demonstrated the observed synergistic effect between calcium ions and the dipeptide YR on the proliferation of human osteoblasts [8]. Given the presence of various metal elements during the mineralization step in the bone matrix [20], we investigated the effect of different metal ions in the presence of dipeptide YR on osteoblast proliferation in this study. Osteoblast growth was measured in the presence of 0.45 μM dipeptide YR and 3.6 μM various metal ions (Figure 1). Notably, among the tested elements, including CaCl_2_, MgCl_2_, CuSO_4_, MnCl_2_, FeSO_4_, ZnSO_4_, SrCl_2_, and Na_2_SeO_3_, the addition of both CaCl_2_ and SrCl_2_ resulted in an additional 12.3% osteoblast growth in the presence of dipeptide YR. It is noteworthy that the calcium and strontium content in human bones far exceeds that in deer antlers or tortoiseshell. Both calcium and strontium belong to the alkaline earth metals in the periodic table and possess similar molecular orbital orientations. It seems likely that strontium ions can efficiently utilize the same calcium channel triggered by dipeptide YR. Furthermore, MnCl_2_ and ZnSO_4_ also exhibited the ability to enhance cell growth numbers in the presence of dipeptide YR, showing an increase of approximately 5.1% and 6.5%, respectively. Conversely, the introduction of CuSO_4_ and Na_2_SeO_3_ resulted in a reduction of osteoblast growth by 18.0% and 12.2%, respectively. As indicated in Table 1, due to the extremely low levels of copper, manganese, and selenium ions in human bone (near or below 1 mg/kg), the impact of deer antler peptide on these ions may be negligible.

### 2.2. Effect of Deer Antler Peptides on Skeletal Muscle Cell Proliferation

**Figure 2:**
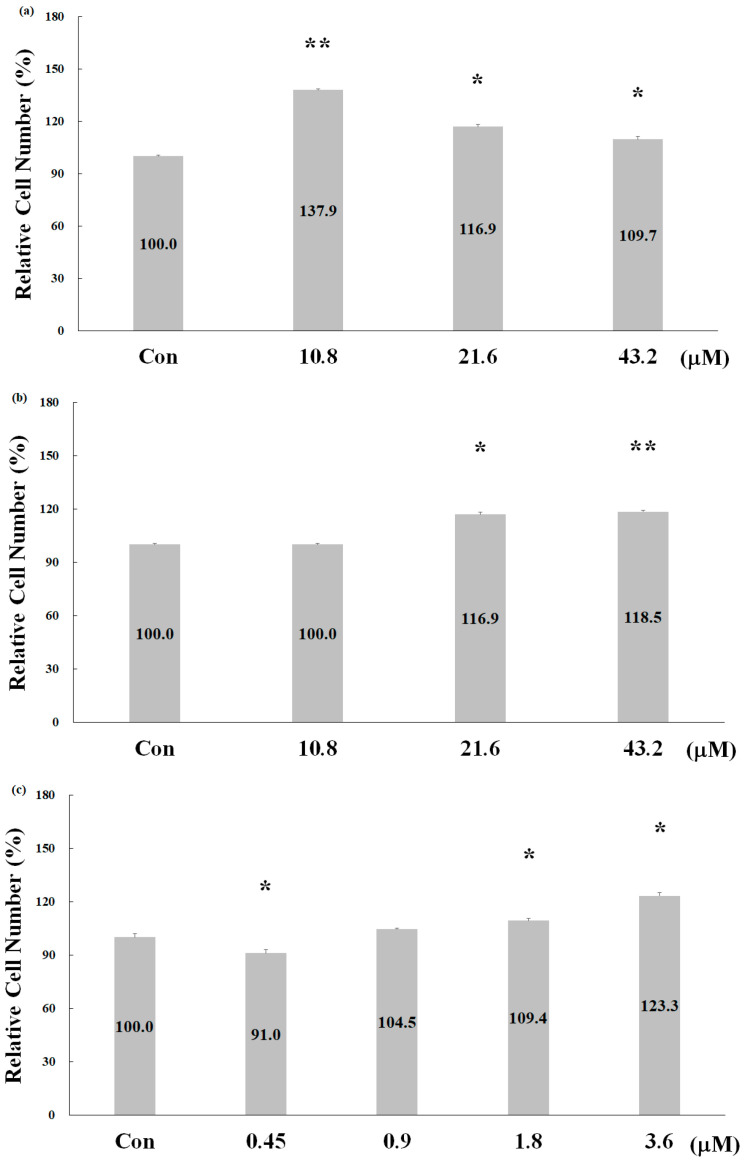
Effects of deer antler peptides on skeletal muscle cell proliferation after 24 h treatment. (**a**) P 5, (**b**) P 3, and (**c**) P 2. Note: Data are presented as means ± SD in three independent experiments. Significance was determined through a two-tailed test analysis, * *p* < 0.05 and ** *p* < 0.005 indicate significant differences between different peptide concentrations treatments.

Because the muscular and skeletal systems collaborate to facilitate movement, we assessed the proliferation-stimulating effects of the three deer antler peptides on myoblasts. Cell numbers were counted after a 24 h treatment with the peptides. As depicted in Figure 2, treatment with peptides 5, 3, and 2 resulted in a 37.9%, 18.5%, and 23.3% increase in C_2_C1_2_ cell numbers in the presence of 10.8 μM peptide 5, 43.2 μM peptide 3, and 3.6 μM peptide 2, respectively. Perhaps not surprisingly, these findings indicate that these deer antler-derived peptides also possess the ability to stimulate the proliferation of muscle cells. This activity is also aligned with the vitality enhancement, one of the documented efficacies of TDAG.

### 2.3. Biological Activities of Deer Antler Peptides on Chondrocyte Growth

**Figure 3:**
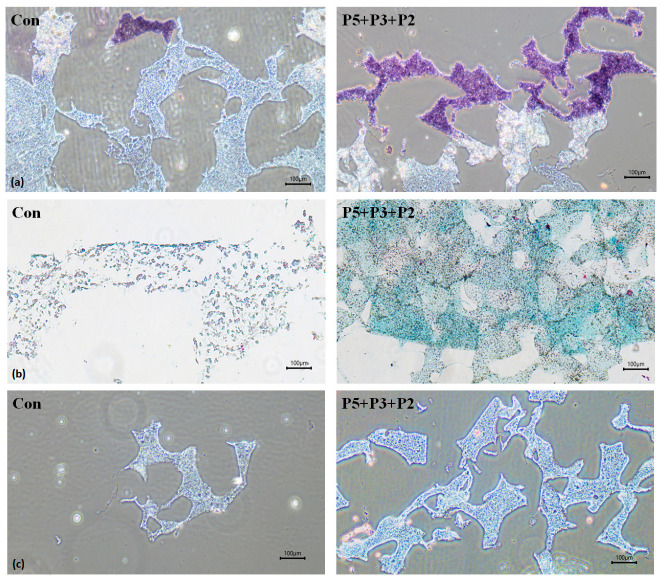
Representative images of histochemical staining for the chondroplug device after the treatment with deer antler peptides for 3 days. (**a**) H&E staining; (**b**) Alcian blue staining; (**c**) Masson’s trichrome staining. Chrondroplug with C20A4 chondrocytes were treated with medium only (Con) or 0.45 μM P5, P3, and P2 (P5 + P3 + P2) for 3 days. (Light microscopy, 40×).

Chondroplug (BioGend Therapeutics LTD, Taipei, Taiwan) is a biodegradable scaffold implant used for cartilage tissue regeneration and the repair of damaged cartilage through implant surgery. Human chondrocytes C20A4 were cultured within Chondroplug in a six-well plate. Following a 3-day treatment of 0.45 μM P5, P3, and P2, the scaffold underwent histochemical staining, as depicted in Figure 3. The application of deer antler peptides significantly enhanced chondrocyte growth, which is evident through H&E staining (Figure 3a). Alcan blue dye reacts with sulfate groups commonly present in glycosaminoglycans found in cartilage. It is apparent that the application of deer antler peptides led to a notable increase in glycosaminoglycan content on the chondrocyte surface, as shown in Figure 3b. Meanwhile, Masson’s Trichrome staining, a widely utilized technique for visualizing collagen fibers, revealed that the treatment with deer antler peptides enhanced collagen synthesis in the vicinity of the chondrocyte matrix, as depicted in Figure 3c.

### 2.4. Chondrocyte Growth in 3D Cell Encapsulation

Chondroplug (BioGend Therapeutics LTD, Taipei, Taiwan) is a biodegradable scaffold implant used for cartilage tissue regeneration and the repair of damaged cartilage through implant surgery.

**Figure 4:**
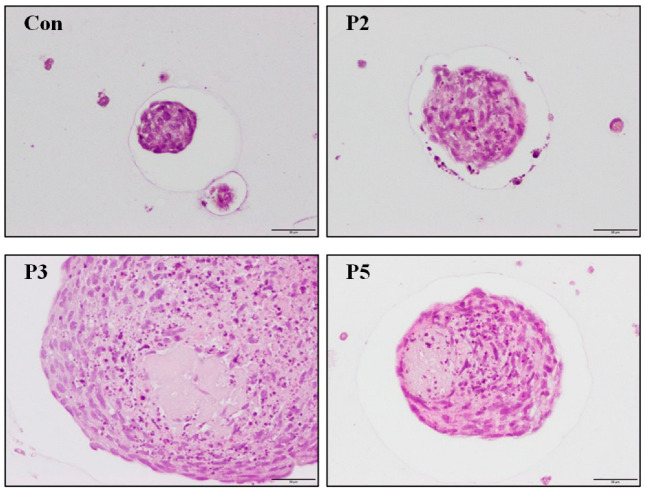
H&E staining for cell encapsulation embedded with chondrocyte C20A4. Cell encapsulation was treated with either medium only (Con) or with 0.9 μM of peptide 5 (P5), 0.45 μM of peptide 3 (P3), and 1.8 μM of peptide 2 (P2) for 28 d (Scale bar size: 50 μm).

In a 3D culture of chondrocytes, the environment encourages the formation of organized tissue structures, including the production of essential extracellular matrix components such as collagen and proteoglycans [21]. Cell encapsulation of chondrocytes is a three-dimensional culture method designed to mimic articular cartilage. C20A4 chondrocytes were entrapped within a permeable polymeric material and then treated with deer antler peptides or culture medium alone. After 4 weeks of treatment, both histological examinations with H&E staining and gene expression by quantitative PCR were employed to assess chondrocyte growth and the underlying signal transduction mechanisms. The results of the H&E stain are presented in Figure 4, showing that treatment with deer antler peptides stimulated chondrocyte growth. Apparently, the size of the encapsulated beads was more significant than that in the control group, suggesting that deer antler peptides can stimulate chondrocyte growth and the production of extracellular matrix components.

**Figure 5:**
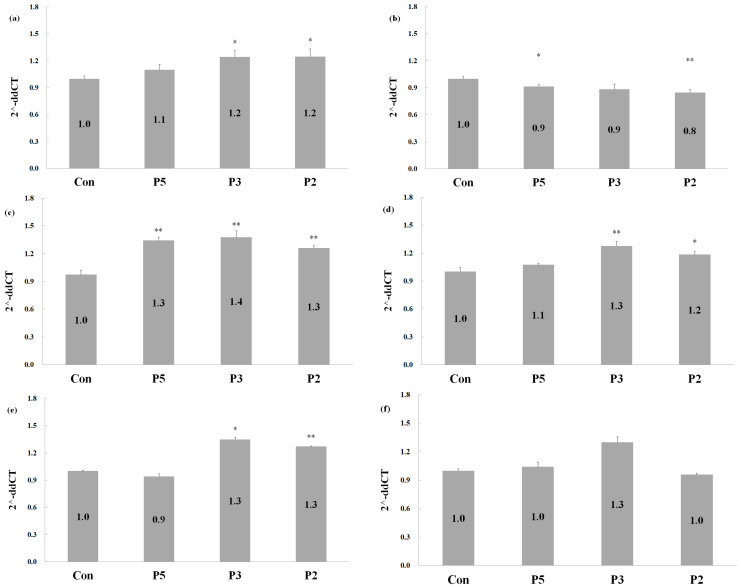
Quantitative PCR for gene expression in cell encapsulation embedded with chondrocyte C20A4 cells after treatment of peptides. Cells were treated with 0.9 μM P5, 0.45 μM P3, and 1.8 μM P2 for 28 d. (**a**) HIF1A, (**b**) SOX9, (**c**) ACAN, (**d**) Collagen I, (**e**) Collagen II, and (**f**) Collagen III. Two-tailed test analysis revealed * *p* < 0.05 and ** *p* < 0.005 for different peptide treatments versus the control.

Further analysis of the signal transduction mechanism was conducted using real-time PCR to evaluate the expression of *HIF1A*, *SOX9*, *ACAN*, *COL1A1*, *COL2A1*, and *COL3A1*. In chondrogenesis, hypoxia-inducible factor 1-alpha (HIF1A) and Sox9 (SOX9) function as potent activators of chondrocyte-specific enhancers for collagen II (COL2A1) and aggrecan (ACAN). Collagen type II, also known as cartilage collagen, is essential for chondrocyte synthesis and is predominately present in cartilage tissue. *SOX9*, *COL2A1*, and *ACAN* serve as specific marker genes for chondrogenesis. RNA was extracted from the encapsulation beads, and the qPCR results revealed that peptides 5, 3, and 2 induced *HIF1A* expression by approximately 1.1, 1.2, and 1.2-fold, respectively (Figure 5a). Notably, *SOX9* expression was slightly inhibited (Figure 5b), suggesting the involvement of other transcription factors in chondrogenesis. Additionally, the expression of downstream genes, such as *ACAN* and *COL2A1*, was evaluated. Deer antler peptides exhibited greater efficacy in upregulating *ACAN* expression, with increases of approximately 1.3, 1.4, and 1.3-fold under treatment with peptides 5, 3, and 2, respectively (Figure 5c). Peptides 3 and 2 also induced *COL1A1* (Figure 5d) and *COL2A1* (Figure 5e) expression. Concerning *COL3A1*, only peptide 3 enhanced its expression (Figure 5f). In brief, deer antler peptides have the potential to enhance the repair of damaged cartilage by regulating the expression of *HIF1A*, *ACAN*, *COL2A1*, and other relevant factors.

### 2.5. Effects of Deer Antler Peptides on Homeostasis between Osteoclasts and Osteoblasts

Bone remodeling is closely related to the homeostasis between osteoclasts and osteoblasts. Osteoblasts release RANKL, M-CSF, OPG, WNT5A, and WNT16 to stimulate the formation of osteoclasts. By contrast, osteoclasts secrete SEMA4D, CTHRC1, C3, and S1P to promote the formation of osteoblasts [22]. Accumulating evidence suggests that excessive intracellular reactive oxygen species (ROS) generation can hinder osteoblast differentiation and expedite bone resorption by osteoclasts [23,24,25]. Excessive ROS production disrupts bone homeostasis by interfering with the differentiation of both osteoclasts and osteoblasts. To address this concern, we examined changes in intracellular ROS concentrations in RAW264.7 cells following RANKL treatment, with or without deer antler peptides.

**Figure 6:**
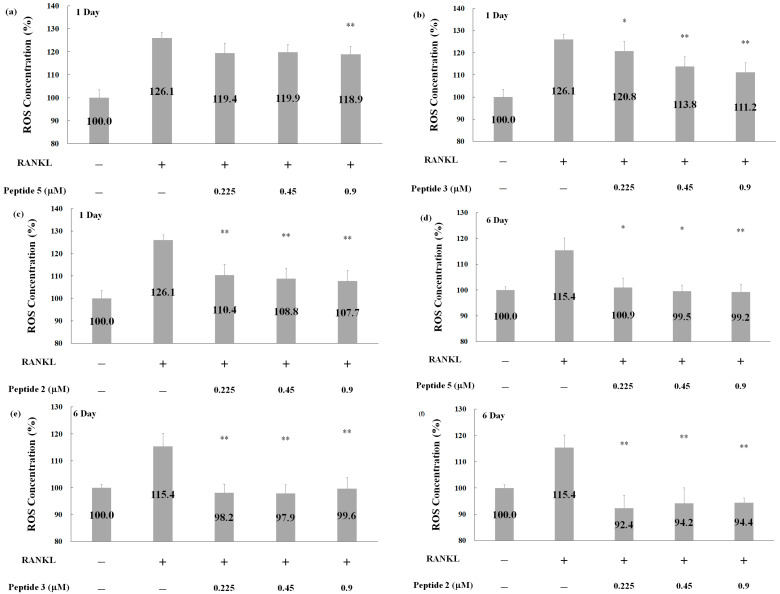
Detection of ROS generation for RAW264.7-differentiated osteoclast after peptide treatment. RAW264.7 cells were treated with RANKL combined with different concentrations of Peptide 5 (**a**,**d**), Peptide 3 (**b**,**e**), Peptide 2 (**c**,**f**), or co-treatment with Peptides 5, 3, and 2 (**a**–**f**) as denoted. ROS concentrations were evaluated after treatment for 1 day (**a**–**c**) and 6 days (**d**–**f**). Through two-tailed test analysis, * *p* < 0.05 and ** *p* < 0.005 between RANKL treatment with peptides versus RANKL treatment only.

Receptor activator of nuclear factor-B ligand (RANKL) directly influences osteoclast precursors in RAW264.7 cells, inducing their differentiation into multinuclear bone-resorbing cells [26]. Therefore, RAW264.7 cells were induced by RANKL to produce osteoclasts in this study. As shown in Figure 6a–c, RANKL treatment for one day increased ROS generation by approximately 26.1% compared to that in the control group. When RANKL was combined with peptides 5, 3, or 2, ROS concentrations generally exhibited a dose-dependent reduction. Notably, 0.9 μM of peptide 2 exhibited the most significant inhibition of ROS production, lowering it by up to 18.4% compared to that by RANKL treatment alone.

Continuing RANKL treatment without peptides for 6 d resulted in maintaining the ROS concentrations at approximately 15.4% higher than that in the control group (Figure 6d–f). Combining RANKL with peptide 2 consistently led to a substantial reduction in ROS concentration, ranging from 21% to 23% (Figure 6f). These results indicate the potential of peptides to inhibit osteoclast differentiation and prevent excessive ROS generation.

**Figure 7:**
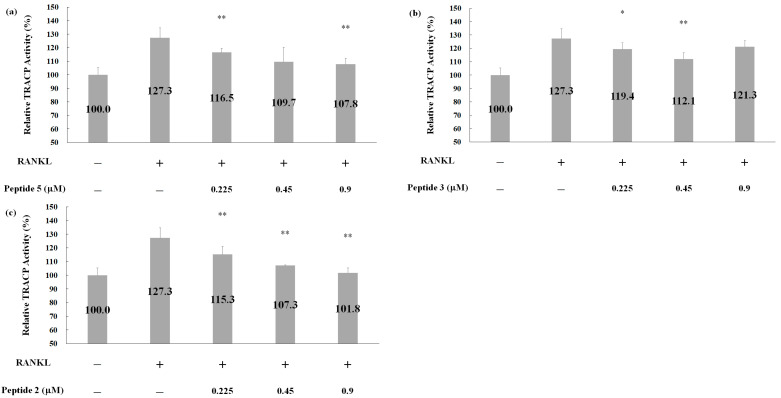
TRACP activity assay for RAW264.7-differentiated osteoclast after peptide treatment. RAW264.7 cells were treated with RANKL combined different concentrations of Peptide 5 (**a**), Peptide 3 (**b**), Peptide 2 (**c**), or co-treatment with Peptides 5, 3, and 2 (**a**–**c**) as indicated. TRACP activities were evaluated after a 6-day treatment. Two-tailed test analysis was conducted, indicating statistical significance with * *p* < 0.05 and ** *p* < 0.005 for comparisons between RANKL combined with peptides versus RANKL treatment only.

Tartrate-resistant acid phosphatase (TRACP) activity serves as an indicator of osteoclast activity [27,28]. Therefore, we assessed whether peptides could prevent RANKL-triggered osteoclast formation, as illustrated in Figure 7. Unquestionably, RANKL induced TRACP activity. However, the combination of RANKL with peptide 5 (Figure 7a), peptide 3 (Figure 7b), or peptide 2 (Figure 7c) treatments effectively inhibited TRACP activity in a dose-dependent manner. The most substantial inhibitory effects on TRACP activity were observed with peptide 5 (0.9 μM), peptide 3 (0.45 μM), and peptide 2 (0.9 μM), resulting in reductions of 19.5% (Figure 7a), 15.2% (Figure 7b), and 25.5% (Figure 7c), respectively. These results unequivocally demonstrate that deer antler peptides can significantly repress RANKL-triggered osteoclast differentiation, leading to a higher inhibition of TRACP activity.

**Figure 8:**
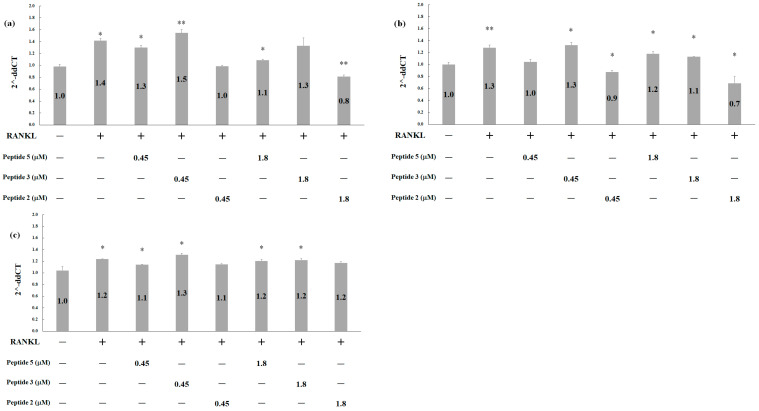
Quantitative PCR for gene expression in RAW264.7 cells after treatment with RANKL, with or without peptides. Cells were treated with 10 ng/mL RANKL, with or without peptides 5, 3, and 2 for 6 d. The working concentration of peptides is denoted in the figures, and the unit of concentration used is μM. (**a**) MMP-9, (**b**) CTSK, and (**c**) DC-STAMP. Statistical analysis was performed using a two-tailed test, with * *p* < 0.05 and ** *p* < 0.005 indicating significance compared to the control for different peptide treatments.

MMP-9, a member of the matrix metalloprotease family, exhibits heightened expression in osteoclasts and plays a pivotal role in bone mineral matrix degradation during bone resorption [29]. As depicted in Figure 8a, RANKL induction led to an increase in MMP-9 expression. However, this phenomenon was effectively attenuated in a dose-dependent manner when RANKL was co-administered with either peptide 5 or peptide 2. Simultaneous treatment of RANKL with 1.8 M of peptide 2 resulted in a remarkable inhibition of MMP-9 expression, resulting in a reduction of approximately 40%.

Another gene specific to osteoclasts is *CTSK*, encoding the cathepsin K protein, which mediates the formation and activation of mature osteoclasts [30]. In Figure 8b, RANKL treatment upregulated CTSK expression by approximately 30%. However, when RANKL was combined with 0.45 μM peptide 5 or 1.8 μM peptide 3, CTSK expression was substantially reduced by approximately 30% or 15%, respectively. Simultaneous treatment with 10 ng/mL RANKL and peptide 2 resulted in a dose-dependent manner effect. Notably, CTSK expression in the presence of 1.8 μM peptide 2 was inhibited as much as 50%.

Dendritic cell-specific transmembrane protein (DC-STAMP) serves as a key regulator of osteoclast cell-cell fusion [31]. As shown in Figure 8c, RANKL treatment resulted in a slight upregulation of DC-STAMP expression. Notably, co-administration of RANKL with 0.45 μM of either peptide 5 or peptide 3 resulted in an inhibition of DC-STAMP expression by approximately 10%. These findings highlight the efficacy of peptide 5, peptide 3, and peptide 2 in effectively suppressing osteoclast formation and activation by mediating the expression of MMP-9, CTSK, and DC-STAMP genes, consequently leading to a heightened level of inhibition.

## 3. Materials and Methods

### 3.1. Materials and Cell Culture

Deer antler peptides, synthesized by Mission Biotech (Taipei, Taiwan), were employed in the study. Human osteoblasts (hFOB1.19), sourced from the American Type Cell Collection (ATCC), and articular chondrocyte C20A4, obtained from MERCK (Darmstadt, Germany), were cultured as reported previously [8]. The cells were incubated at 34 °C in a 5% CO_2_ atmosphere [32]. Mouse skeletal muscle cell lines (C2C12 myoblasts), generously provided by MD Yu-Jen Chiu (Surgery Department, Taipei Veterans General Hospital), were cultured in DMEM high glucose medium with 10% FBS. The C2C12 mouse myoblast cell line was cultured in high glucose DMEM with 10% cosmic calf serum.

### 3.2. Cell Proliferation Assessment

To evaluate the cell proliferation potential of deer antler peptides, hFOB1.19 or C2C12 myoblasts were seeded in a 24-well plate at a density of 4 × 10^4^ cells per well. Subsequently, these cells were incubated with dipeptides (P2, YR) with or without the presence of various metal ions, the concentrations of which are specified, for 24 h. Afterward, the cells underwent a brief 2-min trypsin treatment and were manually counted using a hemocytometer.

### 3.3. Cell Encapsulation

Approximately 1.0 × 10^6^ cells/mL of C20A4 chondrocyte suspension were encapsulated in alginate beads by mixing with a three-fold volume of 1.25% alginic acid solution containing 20 mM HEPES, pH 7.4, and 150 mM sodium chloride, at room temperature [33]. Subsequently, the cell–alginate solution (40 μL) was dropped into a solution containing 10 mM HEPES, pH 7.4, and 102 mM CaCl_2_. After 5–10 min incubation, the cell-encapsulated beads were polymerized and placed into individual wells of a 24-well plate following C20A4 culture conditions. The culture medium, with or without peptides, was changed every 2 d until the cell-encapsulated bead constructs were cultured for 4 weeks.

### 3.4. Reverse Transcription and Quantitative PCR 

C20A4 cells were treated with various peptides for 28 d. The optimal peptide concentration was reported previously [8]. RNA isolation was conducted using the Gene-spin Total RNA purification Kit (Protech Technology Enterprise Co., Ltd., Taipei, Taiwan), followed by reverse transcription with the MMLV Reverse Transcription Kit (Protech Technology Enterprise Co., Ltd., Taipei, Taiwan). All experimental steps strictly adhered to the manufacturer’s instructions. The primers used are listed in Table 2, and the β-actin mRNA levels were used to normalize the mRNA expression levels of the target genes.

### 3.5. Detection of ROS Generation 

RAW264.7 cells were seeded in a 96-well plate (0.5 × 10^4^ cells/well). The cells were treated with 10 ng/mL RANKL, both with and without the addition of deer antler peptides, for durations of 1 d and 6 d. Following treatment, the cells were resuspended in 200 μL of PBS per well. Subsequently, 100 μM 2′,7′-dichlorodihydrofluorescein diacetate (H2DCF-DA) purchased from Sigma was added to each well and incubated for 1 h. Finally, the absorbance of the culture medium was measured with an excitation wavelength maximum of 492 nm and an emission maximum of 520 nm [42].

### 3.6. TRACP (Tartrate-Resistant Acid Phosphatase) Activity Assay 

The TRACP activity assay used the TRACP & ALP Assay Kit from Takara Bio Inc. (Shiga, Japan). RAW264.7 cells were cultured in a 96-well plate (5 × 10^5^ cells/well) for 6 d. Following the removal of the culture supernatant, the cells were resuspended in 0.9% normal saline. All procedural steps adhered to the manufacturer’s instructions. Subsequently, 50 µL of extraction solution was added to each well and incubated for 15 min at 37 °C in a 5% CO_2_ atmosphere. Next, 50 µL of substrate solution was loaded to each well and further incubated for 60 min at 37 °C. Finally, 50 µL of 0.5 N NaOH was introduced into each well to terminate the reaction. After color formation, the absorbance of the solution at 405 nm was measured [43].

## 4. Conclusions

Tortoiseshells served as a historical source of calcium ions. Notably, both calcium and strontium ions stimulated osteoblast growth after a 24-h incubation in the presence of dipeptide YR. Accordingly, the contents of both metal ions in human bone are much higher than those in tortoiseshell or deer antlers. This experiment demonstrates that deer antler peptides can enhance the bio-absorption efficiency of calcium and strontium ions, suggesting a potential clinical improvement for osteoporosis-related issues.

Our research, outlined in this paper, has unveiled that deer antler peptides can stimulate chondrocyte growth and enhance the synthesis of glycosaminoglycans and collagen fibers. These components collectively form a protective matrix, providing cushioning against external shocks and aligning with their potential efficacy in osteoarthritis treatment. Therefore, the activities of these deer antler peptides on chondrocytes contribute to the potential efficacy in managing osteoarthritis.

In this study, we highlight the diverse biological activities of deer antler-derived peptides in various aspects of bone metabolism, such as inhibition of the differentiation of osteoclasts, the cells responsible for bone resorption. Collectively, the aforementioned results presented in this paper establish a scientific foundation supporting the potential of this traditional remedy in effectively treating osteoporosis and osteoarthritis. In addition to these in vitro experimental outcomes, ongoing in vivo animal model experiments aim to further substantiate the efficacy of these deer antler peptides in the context of osteoarthritis and osteoporosis. Safety is consistently a significant concern with most new drugs. However, deer antler peptides apparently do not exhibit this issue. Consequently, these findings could shape future treatment approaches or contribute significantly to developing new therapies for musculoskeletal conditions.

## Figures and Tables

**Table 1:** Comparative analysis of metal element content in human bone, tortoiseshell, and deer antler.

Metal	Human Bone (mg/Kg)	Tortoiseshell (mg/Kg)	Deer Antler (mg/Kg)	Fold *
Ca	171,400 ± 4050	1841.6 ± 33.1	1902.1 ± 33.3	**approximately 90**
Mg	2139 ± 38	429.7 ± 9.0	268.4 ± 8.9	approximately 5–8
Sr	291 ± 20	10.5 ± 0.4	8.0 ± 0.4	**approximately 28–36**
Fe	140 ± 11	19.1 ± 0.8	23.7 ± 0.9	approximately 6–7
Zn	92.8 ± 1.5	22.8 ± 0.5	23.9 ± 1.1	approximately 4
Cu	1.35 ± 0.22	0.39 ± 0.02	0.3 ± 0.02	approximately 4
Mn	0.354 ± 0.004	0.83 ± 0.03	0.29 ± 0.02	approximately 0.4–1
Se	<0.06	0.075 ± 0.004	0.018 ± 0.001	approximately 1–3

* Fold represents the ratio of metal element content in human bone compared to that in tortoiseshell or deer antler.

**Table 2:** Primer sequences for RT-qPCR of target genes.

Gene	Primer Sequence (5′→3′)	Reference
*HIF1A*	FORWARD: TGCTTGGTGCTGATTTGTGA REVERSE: GGTCAGATGATCAGAGTCCA	[34]
*SOX9*	FORWARD: CCCCAACAGATCGCCTACAG REVERSE: GAGTTCTGGTGGTCGGTGTAGTC	[35]
*ACAN*	FORWARD: CACCTCCCCAACAGATGCTT REVERSE: GGTACTTGTTCCAGCCCTCC	[36]
*Collagen I*	FORWARD: TGGTGGTTATGACTTTGGTTACGAT REVERSE: TGTGCGAGCTGGGTTCTTTCTA	[37]
*Collagen II*	FORWARD: TGCTGCCCAGATGGCTGGAGGA REVERSE: TGCCTTGAAATCCTTGAGGCCC	[38]
*Collagen III*	FORWARD: ATGGTTGCACGAAACACACT REVERSE: CTTGATCAGGACCACCAATG	[39]
*β-actin*	FORWARD: AGAGCTACGAGCTGCCTGAC REVERSE: AGCACTGTGTTGGCGTACAG	[40]
*MMP-9*	FORWARD: AGTTTGGTGTCGCGGAGCAC REVERSE: TACATGAGCGCTTCCGGCAC	[41]
*CTSK*	FORWARD: GGCCAACTCAAGAAGAAAAC REVERSE: GTGCTTGCTTCCCTTCTGG	[41]
*DC-STAMP*	FORWARD: TCCTCCATGAACAAACAGTTCCAA REVERSE: AGACGTGGTTTAGGAATGCAGCTC	[41]
*GAPDH*	FORWARD: AACTTTGGCATTGTGGAAGG REVERSE: ACACATTGGGGGTAGGAACA	[41]

## Data Availability

Data will be made available on request.
